# A Fuzzy Shell for Developing an Interpretable BCI Based on the Spatiotemporal Dynamics of the Evoked Oscillations

**DOI:** 10.1155/2021/6685672

**Published:** 2021-04-09

**Authors:** Anna Lekova, Ivan Chavdarov

**Affiliations:** Institute of Robotics, Bulgarian Academy of Sciences, Acad. Georgi Bonchev Str., Bl. 2, PO Box 79, Sofia 1113, Bulgaria

## Abstract

Researchers in neuroscience computing experience difficulties when they try to carry out neuroanalysis in practice or when they need to design an explainable brain-computer interface (BCI) with quick setup and minimal training phase. There is a need of interpretable computational intelligence techniques and new brain states decoding for more understandable interpretation of the sensory, cognitive, and motor brain processing. We propose a general-purpose fuzzy software system shell for developing a custom EEG BCI system. It relies on the bursts of the ongoing EEG frequency power synchronization/desynchronization at scalp level and supports quick BCI setup by linguistic features, ad hoc fuzzy membership construction, explainable IF-THEN rules, and the concept of the Internet of Things (IoT), which makes the BCI system device and service independent. It has a potential for designing both passive and event-related BCIs with options for visual representation at scalp-source level in response to time. The feasibility of the proposed system has been proven by real experiments and bursts for *β* and *γ* frequency power have been detected in real time in response to evoked visuospatial selective attention. The presence of the proposed new brain state decoding can be used as a feasible metric for interpretation of the spatiotemporal dynamics of the passive or evoked neural oscillations.

## 1. Introduction

An EEG-based brain-computer interface (BCI) uses an electrophysiological monitoring method to measure the scalp electrical potentials resulting from ionic currents within the neurons of the brain. By placing multiple electrodes on the scalp, the brain signals correlated with user's emotions and intentions can be registered, featured, classified, and translated into artificial commands for control or communication with the digital devices and services around. The stages of pattern recognition and classification in BCIs call for elements of Artificial Intelligence (AI). Because humans are involved, interpretable and explainable Artificial Intelligence (XAI) [1] needs to be included in the system design. Interpretability means that the cause and effect can be easily determined in the machine learning (ML) models. XAI is a new trend of AI to explain the black-box approaches in ML by context-specific methods in order to make humans understand the reasoning behind their predictions and the errors they make.

BCI community is a multidisciplinary research field where neuroscientists, biomedical engineers, and computer scientists need to work together. Very often this collaboration is impossible and researchers experience difficulties when they try to use the available BCI software tools, such as BCILAB [2], EEGLAB [3], OpenVibe [4], BCI2000 [5], Neuromore [6] and other tools surveyed in [7]. These general-purpose software applications aid the design and testing of both passive and event-related BCIs in different applications. BCILAB [2], an open source MATLAB-based toolbox, provides an organized collection of 100 preimplemented methods and method variants. EEGLAB [3] is an open source toolbox for analysis of EEG dynamics by Independent Component Analysis (ICA). The OpenVibe [4] is a user-friendly software for BCI with graphical user drag-and-drop interface. BCI2000 [5] is a general-purpose BCI system for different task specific BCI applicable methods. Some of these tools are optimized for real-time EEG data processing using Python, C++, or MATLAB scripting box for online processing: OpenVibe Acquisition Server [http://openvibe.inria.fr/acquisition-server], BCI2000 Webserver [http://www.bci2000.org], and MatRiver, a MATLAB DataRiver client [7]. Neuromore [6] allows users to connect to biosensors, such as EEG, and is a biodata acquisition, streaming, processing, and visualization software. It has drag-and-drop user interface allowing the users to get different views of the raw EEG data simultaneously. Neuromore is open source and has a cloud-based platform that connects with wearable devices and provides cloud-based collaborative research and cloud data management. The OpenVibe is considered to be the most user-friendly tool and can be used without much programming skills. However, the use of the real-time WebSocket for BCI online operation needs IT skills in client-server programming and is time-consuming. Although these tools are “general-purpose” ones, they lack comprehensible features and models in designing a custom EEG-based BCI and a lot of skills in MATLAB programming and other software languages are required to support brain computations of the neuroscientists. Widely accepted in the experimental science, such as neuroscience, are the research claims to be based on statistical tests [8]. Therefore, testing the null-hypothesis (also known as significance testing) by Analysis of Variation (ANOVA) and multiple comparison statistics is essential to be friendly configured and embedded in the BCIs for neuroresearch in order to support neuroscientists in their experiments.

On the other hand, computer scientists easily use these tools but face difficulties in designing custom BCI applications. The available today portable brain-listening headsets come with accessible brain measuring hardware and the computer scientists commonly use BCIs to control digital devices or services. The task-evoked underlying neural activity needs to be translated into artificial commands and before designing a specific BCI the computer scientists need to find and map the published neuroexpertise into features, patterns, and control actions. It will be helpful if this mapping is human interpretable. Because the expert opinion is involved for classification, the black-box ML models for classification are not sufficient. We support the computer scientists by quick setup of a fuzzy system. Fuzzy rule-based classifiers can be built using expert opinion, data, or both and are considered more intuitive and AI explainable [9].

We seek to close the gap between computer scientists and neuroscientists by providing a general-purpose fuzzy software system shell for designing a real-time operating EEG-based BCI system with ad hoc brain state decoding by linguistic variables and fuzzy sets participating in interpretable fuzzy IF-THEN rules. The decision-making response is based on the neurons involved in a particular neural computation in terms of the trend (derivatives) in the evoked oscillatory rhythms and neuronal assembly at scalp locations over time. One of our goals in the design of the proposed BCI fuzzy shell (BCIFS) was to profile the data analysis of the software: ready-to-use data for post hoc interpretation by ANOVA and multiple comparison statistics for the neuroscientists and post hoc training and optimization of the fuzzy system parameters for the computer scientists. Thus, the neuroscientists can test their hypothesis and perform initial experiments for testing multiple predictions with contradictions in assumptions simultaneously, while the computer users can digitally translate the published neuroscience findings in interpretable IF-THEN rules for fuzzy interpretation of the spatiotemporal dynamics of the passive or evoked oscillatory rhythms. Additionally, the computer scientists have the freedom to exploit the resulting ready-to-use data in MATLAB for post hoc training and optimization of the fuzzy sets, fuzzy membership functions, and the fuzzy rule-based classifier with different ML algorithms.

The rest of the paper is organized as follows: in Section 2, related works and the proposed solutions are presented.  Section 3 summarizes the mathematical background and the detailed description of the proposed BCI fuzzy shell.  Section 4 presents materials and methods.  In Section 5, the results are exposed and discussed. Finally, conclusions follow.

## 2. Current Status, Problem Statement, and the Proposed Solution

### 2.1. Existing Solutions

EEG signals are subject-specific and with nonlinear behaviour. The real-time brain state evaluation during sensory processing, memory, or decision-making is a challenging task. The current status of the BCIs, sensing technologies, and computational intelligence approaches are surveyed in [10–14]. An overview of recently used EEG noninvasive devices can be found in [10, 14].

#### 2.1.1. Feature Extraction Approaches

The most commonly extracted BCI features from time series signals can be derived from time, frequency, time-frequency, and statistical and spatial domains. EEG features are described and compared in detail in [12–14]. Examples frequently extracted from EEG signals temporal features are maximum, minimum, zero-crossing rate, linear regression, interquartile range, absolute integral, and others. Commonly extracted statistical features are median, mean, standard deviation, absolute deviation, root mean square, skewness, kurtosis, histogram, and total energy. Spectral features commonly extracted are total energy, spectral centroid, spread, slope, decrease, variation, while the most used spatial features are Common Spatial Patterns (CSP). Other used BCI features are nonlinear: Lyapunov exponent, Shannon entropy, correlation dimension, detrended fluctuation analysis, recurrence rate, and others.

Most papers focus on the use of temporal and spectral domain characteristics. BCIs can be based on the evoked potentials such as Event-Related Potential (ERP) [15] or based on the power of the EEG rhythms and the spectral density [16]. ERP components reflect brief bursts of neuronal activity, time locked to the eliciting event. However, time-domain interpretation neglects spectral characteristics that may be important to the classifier. In the oscillatory-based BCI, researchers usually decompose the EEG signal into five frequency bands using FFT. They use a specific band as a correlate with a cognitive task, sensory or emotion responses. For instance, *θ* (4–7 Hz) and *α* (8–14 Hz) band rhythms correlate with the brain activities during working memory tasks. Similarly, the use of spectral characteristics for feature extraction lacks temporal characteristics. To overcome these deficiencies, the dynamics of the oscillatory rhythms with time resolution in msec is proposed in the literature. Such frequency-band specific features that reflect the changes of the ongoing evoked oscillatory rhythms are Event-Related Desynchronization (ERD) and Event-Related Synchronization (ERS) [17–22]. Decreases in power from the reference to the activation interval are expressed as negative values (i.e., *α* desynchronization), whereas task-related increases in power (i.e., *β* synchronization) are expressed as positive values [18]. For instance, the changes of the ongoing evoked oscillations for sensory and cognitive processing result in different ERD/ERS. However, ERS/ERD alone cannot feature the functional connectivity (FC) in the human brain, the temporal correlation between the times series from different brain regions [23].

Feature extraction methods also are categorized according to the domain they are derived from. A typical time-domain-based feature extraction approach is the autoregressive modelling. Among frequency-domain-based techniques are Fast Fourier Transform (FFT), Power Spectral Density (PSD), band power, and central centroid. The most widespread time-frequency-based feature extraction approaches are Short-time Fourier Transform (STFT), Continuous Wavelet Transform (CWT), and Discrete Wavelet Transform (DWT). Another powerful feature extraction approach CSP utilizes localized spatial filters and each of them converts brain waves into different domain where the variance of one group is magnified. The feature extraction methods depend on the type of brain processes being captured plus the choice of the BCI system design. Some feature extraction methods are unsupervised such as Principle Component Analysis (PCA). They do not use raw EEG data labelled with features to learn from, focusing on the differences in the data. Other methods like CSP are supervised and require a set of labelled data to determine the specific spatial features. Applying spatial domain feature extraction could reduce significantly the original size of the electrodes under consideration; however, the computational cost of the improved performance is high. Although the effectiveness of the published feature extraction methods has yet to be reported, studies like [13, 24, 25] illustrated that the high-dimensional and noisy nature of EEG may limit the advantage of nonlinear extraction methods over linear ones. Findings in [24] suggested that multiclass complex BCI task discrimination could gain more benefit from analyzing simple and symbolic features such as Time-Domain Parameters (TDP) rather than more complex features such as CSP and Power Spectral Density. Moreover, from the comparison in [24], it was concluded that the complex ones produced only slightly better classification results. Wen et al. in [25] proposed genetic algorithm-based frequency-domain features and proved that they are superior to the nonlinear features in terms of the ratio of interclass distance and intraclass distance.

To sum up, the selection of the EEG electrodes for classification and their features is critical for both the accuracy of the classifiers and the calculation cost. Some features are not cost effective and suitable for operating in real time. For example, the evaluation of Independent Components (ICs) derived by ICA or Common Spatial Patterns (CSP) more often is performed offline and the calculation cost is high. The resulting features are not human interpretable, while the ML models for their extraction are not explainable. Since the more intuitive and human-interpretable features that can be used in both passive and event-related BCIs are ERS/ERD, our starting assumption originated from [18, 20].

#### 2.1.2. Cross-Subject Training

Cross-subject training is another challenge linked with the BCI system. Sometimes, the training is for a long time, which causes users to become fatigued. Motor imagery (MI-)based BCIs need most training, while the most robust performance across users has the BCIs based on steady-state (motion) visually evoked potentials. When the user receives either a motion reversal frequency or fixed frequency of flashing visual stimuli, the potential brain activity produces the same frequency as a response. For instance, user training was not required in the flicker-free Steady-State Motion Visually Evoked Potential (SSMVEP-)based BCI system proposed in [26]. The developed paradigm used new ring-shaped motion checkerboard patterns with oscillating expansion and contraction motions for visual stimuli. The frequency energy of SSMVEPs was concentrative and the visual stimuli evoked “single fundamental peak” responses after FFT signal processing and canonical correlation analysis. This method has shown highly interactive performance as a paradigm of BCIs with zero training. However, authors in [27] questioned “to train or not to train” and although this is a survey on training of feature extraction methods for Steady-State Visually Evoked Potentials (SSVEP-)based BCIs, the main challenges of how to reduce the user training while maintaining good BCI performance were analyzed. The criticism here is that training-less systems are more practical, however with limiting performance due to intersubject variability.

In general, less training and high performance make the ERP-based BCI system widely used. Some recent studies tried to reduce the calibration time with new subject-independent training methods. In [28], authors introduce the concept of a generic model set. They used ERP data from 116 participants to train the generic model set and trained ten models by weighted linear discriminant analysis. The results from testing the validity of the generic model set demonstrated that all new participants matched the best generic model.

PSD- or ERD-based BCIs also need to be trained, either by session-independent or subject-independent training methods. Different methods have been surveyed in [10]; however, the training is either features or application specific. Recently, the Transfer Learning (TL) in EEG decoding showed great potential in processing signals across sessions and subjects, as can be seen from [10, 29]. The principle of TL is to transfer knowledge from different but related tasks using existing knowledge learned from already accomplished tasks to help with new tasks. In order to train the feature extraction or classification model, the large-scale and high-quality datasets are used to obtain strong robustness and high classification accuracy for the new tasks. A lot of case-studies are surveyed in [29] showing how TL improves the cross-subject transfer and the practicality of real-world BCI applications for different features and tasks.

#### 2.1.3. Classification Methods

Regression or classification algorithms could be utilized to identify various brain activity patterns by the BCI system and translated into commands. The most explored machine learning techniques for classification of EEG signals are based on supervised learning, where a model is created from a training set of EEG signal features to its labels. Example algorithms are *k*-nearest neighbor (K-NN) [30–32], support vector machine (SVM) [32–35], naive Bayes (NB) [32], linear discriminant analysis (LDA) [36, 37], convolutional neural networks (CNN) [38], deep belief network (DBN) [39], AdaBoost ensemble learning [40], lattice computing [31], and fuzzy logic-based classifiers [32, 41, 43–46]. Examples of unsupervised learning algorithms are the reinforcement learning (RL), *k*-means, affinity propagation, spectral clustering, hierarchical clustering, and others. Among them, RL is a prominent unsupervised learning algorithm [47]. RL is flexible and general in its applicability and efficiency for real time and personalized learning in a complex stochastic environment that requires control actions to optimize the system parameters. The RL “agent” acts on the digital world through actions and receives rewards to learn what action to undertake within the current situation. RL is less dependent on the quality of the label information that results in high efficiency of data utilization. Q-learning is a model-free RL and does not require a model of the environment. It handles problems with stochastic transitions and rewards. The Q-learning algorithm has a function “Q” that calculates the quality of a state-action combination according to the maximum expected rewards. RL and its variations, such as deep Q-learning, are applied in different BCI contexts and analyze dynamically the EEG data captured in an experiment. The reward in the EEG-based BCI system could be explicitly based on the EEG signals or implicitly based on system-response parameters. EEG response from the BCI system serves as a reward for the RL agent to learn the features or control actions. Some studies are based on the reward prediction error theory of dopamine [48]. Other studies use EEG signal as the error signal underlying mechanisms of the human error processing [49]. In [50], the system performance is the indicator for the reward calculation. In this study, authors introduced deep reinforcement Q-learning to study the correlation between drowsiness and driving performance. Authors indirectly measure the mind states based on indicators measured during the system performance, such as Response Time (RT). RT measures how quickly the subject reacts to a stimulus and yields the reward. RT is used to assess the current action against the current state, which is the EEG data in the current time window. An optimal policy was always assumed and exploited for action selection and the results showed that the trained model could trace the variations of mind state in a satisfactory way against the EEG data. Usually RL measures future reward to assess the current action. The specific of the proposed Q-learning in [50] is that the reward (in terms of RT) is measured in some latency. However, this brings elements of supervised learning, such as the transition weight beta and history-dependent prediction. Therefore, although the RL is intuitive and does not need an extra XAI, the paradigm of RL requires abstraction and instantiation of the agent, environment, state, action, and reward according to the specific of the learning problem. Q-learning is capable of solving problems with limited states and actions; however, in order to evaluate optimal policies, the value function Q needs to be defined precisely and is out of scope of the neuroscientists. More information for deep learning and unsupervised and semisupervised learning algorithms could be found in [12, 14].

A fuzzy rule-based classifier (FRBC) is a fuzzy system specifically configured for performing classification tasks that consists of a number of classification rules and utilizes fuzziness only in the reasoning mechanism of the classifier [51]. FRBC can be built using expert opinion, data, or both. When the FRBC is created directly from numerical data simple heuristic procedures, neurofuzzy techniques, clustering methods, fuzzy nearest neighbor methods, and genetic algorithms can be used [52]. Majority vote based on a single winner rule (the class with the maximum total strength of the vote) usually classifies the new pattern. BCIs with fuzzy rule-based inference for brain states pattern recognition and classification can be found in [41–46, 53]. Almost all reported results proved that fuzzy rule-based classifiers were not necessarily less accurate than other classifiers. Gu et al. in [41] extracted power spectral features that have been labelled for each trial. The classification model consisted of four types of fuzzy rules that determined the finalised predicted label. Five well-known supervised ML classification methods as SVM, K-NN, NB, ensemble for boosting, and discriminant analysis classifier (DAC) had been trained and the comparison results showed outperformance of a fuzzy rule-based classifier. However, the proposed fuzzy model is task specific; i.e., it was applied to classify motor imagery (MI) data in a passive BCI. Nguyen et al. in [45] introduced a multiclass type-2 fuzzy logic (FL) classifier, where fuzzy parameters were trained using a metaheuristic population-based particle swarm optimization algorithm. CSP is used to extract significant features that are then fed as inputs for classification. The proposed model was also applied to MI BCI. The benchmark four-class MI BCI dataset from the BCI competition IV was used in the analysis of the study. The results from experiments showed the great accuracy of the combination of CSP and type-2 FL compared to LDA, NB, K-NN, ensemble learning AdaBoost, and SVM. Bhattacharyya et al. in [44] proposed two types of multiclass classification algorithm by fusing interval type-2 FL and Adaptive-Network-based Fuzzy Inference Systems (ANFIS). The experimental results showed that the proposed algorithms performed better than LDA, SVM, and NB when dealing with uncertain EEG data. Das et al. in [46] also proposed an interval type-2 fuzzy system using extended Kalman filter based learning algorithm. The BCI competition data of MI type was used in the analysis of the study and the performance evaluation of the FL model showed higher accuracy than SVM, as well as several other fuzzy systems including the evolving fuzzy rule-based classifier, online sequential ANFIS, metacognitive neurofuzzy inference system, and metacognitive interval type-2 fuzzy system. Tsai et al. in [53] proposed a Takagi-Sugeno fuzzy neural network-based algorithm with single-channel EEG signal for the discrimination between light and deep sleep stages and reported high accuracy in the classification.

To sum up, the proposed FRBC models are brain headset or application specific. Some of them use black-box machine learning approaches; however, in a combination with fuzzy rules, a reasoning can be performed in order to argue the inner logic of the classifiers. The combination of antecedents and consequences persists in the discipline of argumentation [1]. Although the interpretability and accuracy are considered to be contradictive requirements, the recent tendency is to increase the explainability without hurting model performance much. The authors in [54] proposed a fuzzy classifier by a combination of rule granulation and rule consolidation methods. They obtained the maximum possible classification accuracy with as simple classifier as possible and the method offers the possibility of finding a good compromise between interpretability and performance. To the best of our knowledge, this classifier is not yet implemented in the BCI research; however, the cross-validation results in [54] were prominent. They did not confirm the frequent claim that “Naive Bayes often outperforms more sophisticated classification methods.” On nine benchmark datasets and four classifiers, the naive Bayes one appeared to be winner only in three cases, while the proposed method won in four.

### 2.2. Problem Statement

Summarizing the bibliography research, both feature extraction and classification of EEG data are brain headset or application specific and depend on the custom BCI task. For instance, the highly interactive performance of the evoked-related BCIs proposed in [26] cannot be applied for developing passive BCIs that do not rely on external stimuli, such as emotion recognition, mental workload assessment, or driver drowsiness. Features extraction techniques and models training are not always human interpretable and often are offline. Studding the temporal correlation between the EEG time series from different brain regions in the human brain is not supported online. The big EEG data impose large feature dimensions, extensive set of training data, and machine learning models with a black-box approach. Some ML models perform better than others; however, they are harder to explain. Users want to trust the systems that they are using and to know why a model comes up with the predictions they output. This led to significant growth of XAI over the last few years; however, XAI is an extra work and sometimes it may be difficult to find the cause.

Considering the problems above, we define the requirements to be searched for as follows:

- A framework that can adaptively support a wide range of EEG-based BCI applications
- New brain states decoding with more understandable interpretation of the spatiotemporal dynamics of neuronal activations and neuronal assembly
- Explainable classification model with interpretable linguistic features to support the development of practical BCI applications
- Traceable and comprehensible process of class prediction
- Ubiquitous EEG-based BCI to operate in real time locally or remotely, on different platforms and EEG devices
- Daily live use with practical artifacts cleaning online, less subject-specific, and with minimal training phase

### 2.3. Proposed Solution

We first searched for a new brain states decoding and for more understandable interpretation of the functional connectivity of the neurons involved in the brain processing. Our starting assumption from [20] is the interpretation of ERD as a correlate with the activated cortical areas with increased excitability. We consider that the rate of the oscillatory rhythms is a steady-state endogenous or exogenous brain process with specific functional significance for the evoked neuronal activation and assembly. Thus, we featured the ERS/ERD within a specific ongoing EEG band power by the bursts in change over a certain time window. We denote the rate of increase/decrease (burst) as ERS”/ERD”. Endogenous and exogenous ERS”/ERD” have difference in latency. We define the latency as the delay between the evoked brain activities. The ERS”/ERD” with a peak latency within 250 to 350 msec reflecting the elicited internal neural processes (endogenous) are consistently observed in various executive or memory tasks. The ERS”/ERD” with a peak latency within 100 to 150 msec are attributable to external stimuli or emotional reactions and are typically associated with sensory systems, e.g., steady-state visual or auditory responses.

In order to observe and interpret the ERS”/ERD” in a human readable way, we use linguistic variables and IF-THEN rules where the second derivatives participate and feature the changes in brain rhythms at scalp locations over time. The ERS”/ERD” are described by linguistic variables and discriminated by fuzzy membership functions. The functional connectivity of all brain regions correlated with the evoked event or stimuli is described in the IF part of the rules by a specific combination of linguistic variables. The proposed technique for developing a fuzzy BCI system is the Sugeno fuzzy model (also known as TSK fuzzy model) [55]. It has been chosen because it has a flexibility in the fuzzy system design. TSK model can be used to generate fuzzy rules from a given input-output dataset and thus to train a fuzzy rule-based classifier. Regarding satisfactory accuracy proposed in [53], a four-layer Takagi-Sugeno fuzzy neural network classifier has been reported in the BCI research field. However, in order to go from the neural network black-box approaches to interpretable and explainable model, we studied and implemented an approach proposed in [54] for simple supervised training of a fuzzy classifier via a combination of rule granulation and rule consolidation methods (RGRC). With a slight modification of the criteria for the rule consolidation, we embedded this method in the proposed BCI system to train a fuzzy classifier offline.

Although a classification is a basic task in EEG pattern recognition, sometimes the fuzzy system has to be used in an operation mode and the brain activity to be characterized by formulas that participate in the consequent part of the fuzzy rules. For instance, the EEG_W score (1) known to be related to cognitive processes like workload, engagement, attention, and fatigue [56] is computed from *N*_*e*_ electrodes, placed in the occipital lobe for visual processing evaluation, in frontal lobe for emotional processing, and in the temporal lobe that processes auditory information.

(1) $$\begin{array}{c} \text{EEG}\_\text{W}=\log\left(\frac{\sum_{i=1}^{Ne}\beta_{i}}{\sum_{i=1}^{Ne}\theta_{i}+\alpha_{i}}\right). \end{array}$$

Another functional relation (2) that evaluates a current emotional state based on high/low arousal and positive/negative valence in [57] is computed from four electrodes, placed in the prefrontal cortex (AF3, AF4, F3, and F4). The associated *β*/*α* ratio is a reasonable indicator of the arousal state of a person, while valence (2) is estimated by computing and comparing *α* and *β* power in the frontal channels F3 and F4.

(2) $$\begin{array}{c} \text{Valence}=\frac{{}_{\alpha }F4}{{}_{\beta }F4}-\frac{{}_{\alpha }F4}{{}_{\beta }F4}. \end{array}$$

In both cases, the TSK fuzzy model can be used as a universal approximator of known functions with specified error bounds with computationally efficient defuzzification process. The crisp output value in TSK model is a mathematical combination of the outputs and the rules strength. The fuzzy membership functions can be defined experimentally or statistically. We automatically built trapezoidal fuzzy membership functions during the baseline phase. This shape is chosen because the upper base of a trapezoid takes care of the small scattering due to the oscillatory nature of EEG that causes false featuring. The mean and standard deviation participate together with several coefficients that are tuned according to the used brain headset.

**Figure 1:**
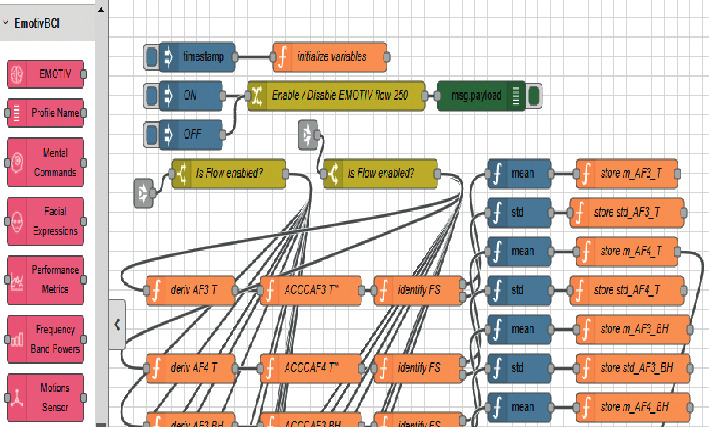
Baseline phase for the frequency bands of interest in a Node-RED flow. Node-RED uses a visual programming for wiring together with code blocks into flows. The connecting input nodes on the left panel are a custom library for integration of the EEG data streams from the EMOTIV EPOC + headset to Node-RED third party software or hardware.

**Figure 2:**
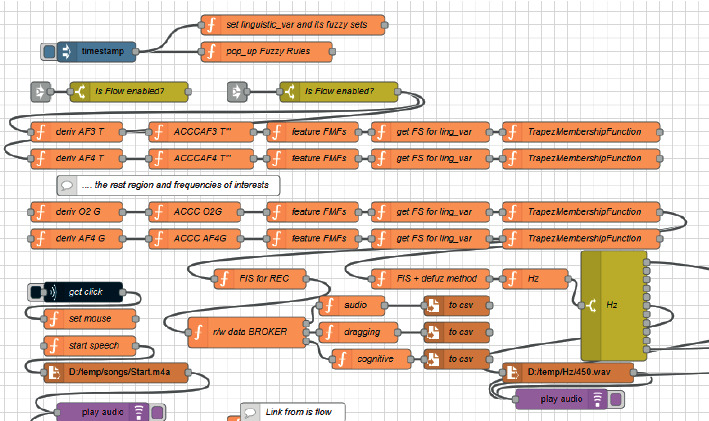
Node-RED flow for EmotivBCI-maze solving task. The dark blue node is a Python block that handles the mouse events for identifying whether the mouse is moving or not.

**Figure 3:**
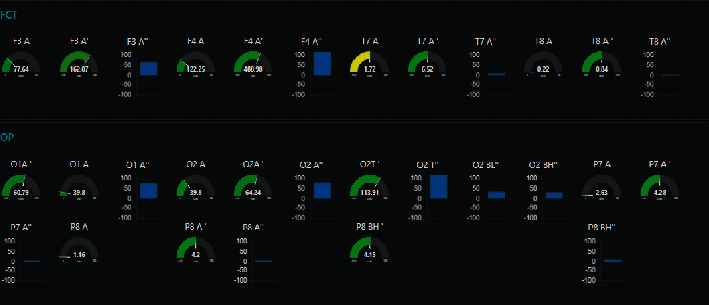
Node-RED front-end graphical user interface, a live data dashboard, where bar and gauge graphs monitor the electrode levels for the brain oscillatory rhythms and their features.

In the proposed BCIFS, we exploited the concept behind the Internet of Things and the Node-RED approach [58] to make all sensing, computation, and memory integrated into a single standalone platform. Node-RED uses a visual programming for “wiring together” of code blocks and make up “flows” to carry out tasks by connecting nodes (input, processing, output, and UI nodes) in a browser-based flow editor (Figures 1 and 2). The described below article's contributions from 5 to 8 are because BCIFS is built in Node-RED, which is a cross platform based on Node.js event-driven model. The flows are stored using JSON and can be easily imported and exported for sharing with others. All these make the device, task, and service independent and portable to operate locally or remotely. The Node-RED standard front-end graphical user interfaces are used for ERS”/ERD” monitoring. It might be observed by a live data dashboard (Figure 3) or heard in the background by passing the result values into a code block for an audio player. The bar and gauge graphs in the dashboard monitor the electrode levels for the brain oscillatory rhythms and their features. For instance, the power of *α* brain oscillation and ERS' values for the electrode O2 are being passed to gauge graphs O2 A and O2 A' (left and centred gauges on Figure 3), while ERS” values are passed to the bar graph O2A”. The colour of the gauge depends on the value being passed into it and changes from the green via yellow to the red corresponding to a change from a reference value to a burst.

The main contributions of this study are the following: (1) a general software system shell for developing both passive and event-related BCIs with quick setup, short training phase, and for real-time application in different contexts, such as executive or memory tasks, sensory processing, neurofeedback, and BCI control; (2) new brain state decoding for human-interpretable feature extraction in terms of burst in change of the neuronal synchronization or desynchronization at scalp-region level; (3) digital twin-based optimization for tuning the parameters of the fuzzy membership functions; (4) practical and real-time artifact collecting and cleaning; (5) easily adapted different EEG-based brain headsets; (6) easily adapted variety of digital devices and services operating in the IoT; (7) real-time analysis of recording EEG rhythms with options for visual or audio representations at scalp-region levels in response to time; (8) remote use of the developed BCI either for operating or for performing experiments; (9) a proof-of-concept: spatiotemporal dynamics of brain connectivity during the evoked visuospatial selective attention.

## 3. Fuzzy Shell for Developing a Custom EEG BCI

The BCIFS is built in the Node-RED platform, taking full advantages of its low-code programming for event-driven applications and wiring together hardware devices, APIs, and online services. The streaming of EEG time series and TSK fuzzy model is developed in a browser-based flow and can run on low-cost hardware such as the Raspberry Pi and in the cloud and IoT. Integrating data streams from different EEG-based headset needs to be done via a custom Node-RED library of input nodes, which allow interfacing the headset technology with other Node-RED nodes.

### 3.1. Featuring of the EEG-Time Series

EEG devices for measuring the ongoing brain activity provide a stream of constantly changing brain time series. Suppose that we are given an EEG dataset denoted by S that contains N number of trials for one subject. Each trial contains EEG records in respect to time and electrode locations belonging to one or several classes (patterns) for the brain activity under consideration. The dataset S is denoted by

(3) $$\begin{array}{c} S=\left\{\left(S_{1},L_{1}\right),\ldots ,\left(S_{i},L_{i}\right),\ldots ,\left(S_{N},L_{N}\right),\right\}, \end{array}$$

where *i* = 1, ..., *N*.

*S*_*i*_ represents the EEG records in *i*^th^ trial. *N* is the number of trials. *L* is a column vector that assigns each trial to one of the associated labels for a single class (*C*). In case of multiple classes, the multilabel classification for *i*^th^ trial is presented as raw vector and its length corresponds to the number of classes *k*.

(4) $$\begin{array}{c} L_{i}=\left\{l_{iC_{1}},\ldots ,l_{iC_{k}}\right\}. \end{array}$$

Each trial has to be present as an input matrix

(5) $$\begin{array}{c} S_{i}\in R_{T\times E}, \end{array}$$

where *E* is the number of electrodes and *T* is the number of time samples per trial. Since the time resolution of the brain signals is in the magnitude of msec and the trial is in the range of sec, time windowing is applied for each trial in order to be more informative for classification. Let *w* denote the size of the window. After referencing the studies in [59–61], we found that *w* is usually 128 msec with no overlapping windows or with overlapping of 5 msec. Experimentally, we defined similar sizes: *w* = 125 msec for exogenous and *w* = 150 msec for endogenous brain processes. The used features (*F*) for categorizing the brain activity reduce the dataspace dimension in the windows. Thus, *S*_*i*_ can be represented as

(6) $$\begin{array}{c} S_{i}\in R_{F_{w}\times E}. \end{array}$$

*F* may be any kind of temporal, statistical, spectral, or nonlinear feature over a certain time window. The window's length defines long-term or short-term interpretation.

The integrated Node-RED third-party software or hardware might be robots, programs for neurofeedback training, serious games, etc. The featured brain electrical activity is translating different type of commands such as robot navigation, touching digital objects on the screen, and switching home. However, no uniform place exists in the brain where a command is stored as a set of neurons. Memory and thinking during the command generation evoke distributed neuronal activity; we can only use approximate reasoning over this EEG activity in order to map them to a digital command. This imposed us to discriminate these brain patterns and describe them by linguistic variables, fuzzy sets, and fuzzy rules according to the location of scalp electrodes and bandwidth. The scalar strength in the premises of the fuzzy rules and the crisp values in the consequences designate the specific functional significance for the evoked neuronal activation and connectivity.

### 3.2. Mathematical Background of the TSK Fuzzy Model

Fuzzy logic takes decisions and recognizes patterns using linguistic variables, “degree of membership,” and fuzzy inference. It maps an input space to an output space using a series of fuzzy IF-THEN rules. Uncertainties are presented as fuzzy sets (*A*_*i*_), which are often expressed by words and interpreted by their membership functions *μ*_*A*_. TSK structure consists of rules in the form

(7) $$\begin{array}{c} R_{i}\text{:IF }x\text{ is }A_{i}\text{ THEN }y_{i}=a_{0}^{i}+\sum_{k=1}^{n}a_{k}^{i}x_{k}, \end{array}$$

where *x*=(*x*_1,_*x*_2_,…, *x*_*n*_) ∈ *S* is a matrix from the brain signal responses at scalp level representing the inputs defined in domain (*S*): *S* ∈ *R*_ERS^″^/ERD_*w*_^″^×*E*_; *A*_*i*_ is a fuzzy set defined on (*S*); *y*_*i*_ is a scalar output corresponding to rule *i*; *a*_*k*_^*i*^ are the consequence parameters associated with rule *i*. For a zero-order TSK model, the output level *y* is a constant. *i* ∈ {1, .., *p*}, where *p* is the number of fuzzy rules.

The simplest fuzzy rule-based classifier is a fuzzy IF-THEN system with a class label staying in the consequences. A fuzzy classifier is constructed by specifying the classification rules:

(8) $$\begin{array}{c} R_{i}:\text{if }x\text{ is }A_{i}\text{ THEN }l_{j{\text{C}}_{1}}, \end{array}$$

where *l*_*j*C_1__ functions as a label.

An example rule is “*IF x*_1_*is A*_1_*AND x*_2_*is A*_2_*THEN* class label is 1.” Such argumentation is easier to be obtained from the neuroscientists. The actual numerical value in the consequences is irrelevant because the class is a nominal variable. All rules “vote” for the class in the consequent part and the majority of these votes discriminate the class; i.e., the maximum aggregation method is applied.

A useful special case for “voting” [51] is the support for each class as a single constant value, usually within the interval [0, 1]:

(9) $$\begin{array}{c} R_{i}:\text{if }x\text{ is }A_{i}\text{ THEN }y_{1{\text{C}}_{1}}\text{ AND}\ldots y_{j{\text{C}}_{1}}\text{ AND}\ldots y_{l{\text{C}}_{1}}, \end{array}$$

where *y*_*j*C_1__ are constants within the interval [0, 1] and *l* is the number of labels for class 1.

In this model, every rule votes for all the classes and the rules are aggregated and defuzzified by using the weighted average:

(10) $$\begin{array}{c} y\left(x\right)=\frac{\sum_{i=1}^{p}\mu_{A_{iw}}\left(x\right)*y_{i}}{\sum_{i=1}^{p}\mu_{A_{i}}\left(x\right)}, \end{array}$$

where *μ*_*A*_*iw*__(*x*) is the degree of fulfillment of *i*-th rule.

(11) $$\begin{array}{c} \mu_{A_{i}}\left(x\right)=\mu_{A_{iw}}\left(x_{1},x_{2},\ldots ,x_{n\prime }\right)=T_{w=1}^{n^{\prime }}\mu_{A_{iw}}\left(x_{f}\right), \end{array}$$

where *n*′ is the number of input variables in *i*-th rule (*n*′ ≤ *n*) and **T** is a type of *t*-(co)norm, such as minimum or product. Since each rule has a crisp output, the overall output is obtained via weighted average, thus avoiding the time-consuming process of defuzzification required in the Mamdani fuzzy model.

First, BCIFS separates the registered EEG data in terms of band average power according to the location of the scalp electrodes. After the preprocessing, BCIFS evaluates variations (derivatives) in ERS/ERD at scalp-source level in response to time. Then, fuzzy reasoning is performed according to the current fuzzy rule base (FRB) and inference. The chosen TSK fuzzy model of zero order uses a set of simple functions that require low CPU memory resources and presents a low time response. Another advantage of this model is that the fuzzy rules can be generated from a given input-output dataset for training a fuzzy rule-based classifier.

### 3.3. Generating the Fuzzy Rule-Based Classifier

During the design of BCIFS, the premise and consequence parameters have to be identified. The electrodes of interest are described by linguistic variables, whereas the rate of change of the evoked oscillatory rhythms is described by fuzzy sets. For instance, following the proposed brain maps of coherence in [62], there is significantly higher coherence at the frontal and right parietal sites for the *θ* band when watching a negative film compared to the neutral state.

 
*IF* “*F*4_*T*_*ERS*”300” *is* “*high*” *and* “*O*2_*T*_*ERS*”300” *is* “*high*” *and* “*P*8_*T*_*ERS*”300” *is* “*high*” *THEN valence is* 0.1.

Here, the output label (valence on the scale [0, 1]) interprets the valence according to the chosen sliding window. ERS”300 means that the rate of change peak (burst) comes with latency of 150 msec over a 300 msec window, shifted every 150 msec.

The FRB is set up according to the significance of the underlying role of each electrode and frequency. Only the critical changes have to be described in the IF-THEN rules. Rules with contradiction in assumptions can be separated in several FRBs working in parallel. We use fuzzy trapezoidal membership functions that are simple and fast for calculation. The upper base of the trapezoid takes care of the small scattering that causes false ERS”/ERD” and wrong baseline recordings during the reference phase. The upper base ignores the wave scattering in the frequency domain, while the legs of the trapezoid eliminate the spikes due to artifacts. When the right leg is perpendicular to the base, the right slope for fuzziness is eliminated and the high bursts produced from artifacts are not evaluated. If we are interested in any “smart artifact,” we can add additional fuzzy membership, so called “artifact,” with appropriate trapezoid parameters. Thus, events related to a facial expression can be classified according to the evoked artifacts.

## 4. Materials and Methods

In this section, the feasibility of the proposed BCI fuzzy shell is illustrated. This was proven by real experiments for evaluating the spatiotemporal dynamics of neural oscillations during the evoked top-down visuospatial selective attention.

### 4.1. Scientific Context

**Figure 4:**
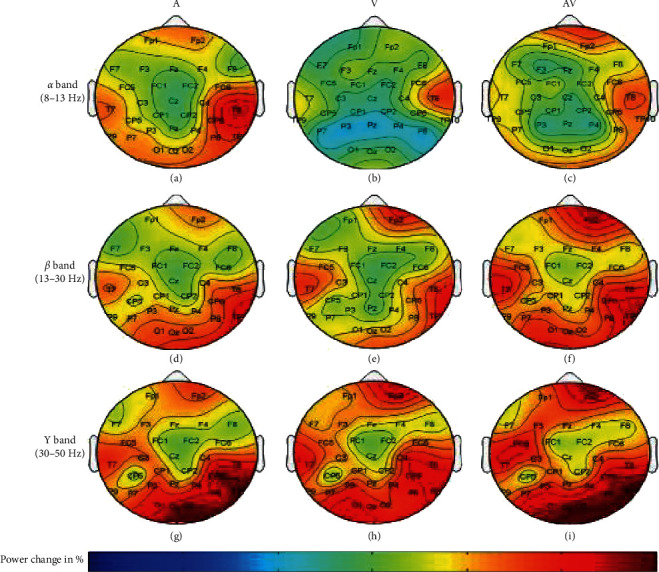
Grand-averaged power change during the attention task compared with the passive view task in *α* band, *β* band, and *γ* band for auditory (A) cues and visual (V) and audiovisual (AV) cues (adapted from [69]).

Attentional processes are the brain's way to cope with the information overload and focus on some stimuli, while suppressing others. Attention is commonly categorized in top-down (or endogenous) attention, an internally induced mental focus on self-thoughts, memories, or abstractions, and bottom-up (or exogenous) attention, an externally induced mechanism that is directed by stimuli from the surroundings. Top-down attention is under voluntary control and is also known as “goal-directed” attention, whereas bottom-up attention is “data-directed.” Endogenous oscillations are attributable to internal neural processes and include a well-known set of frequencies [63]. Exogenous oscillations are driven by the external stimuli and are typically associated with sensory systems, e.g., the auditory steady-state response [64]. Attention in the visual system is extensively studied over the past decades. Visual spatial attention can be either exogenously captured by a salient stimulus that overrides internal goals or can be endogenously allocated by voluntary effort while processing multiple targets [65]. The brain regions actively involved are the prefrontal, parietal, and occipital cortex [59, 60, 66–69]. The prefrontal lobe is thought to be involved in executive functions of the brain: problem solving, judgement, attention, working memory (WM), and motor programming. Many studies have indicated that frontal *θ* activity is closely related to enhanced attention and that sustained neuronal activity is necessary to maintain the WM of representations [22, 70, 71]. Other studies report that *θ* increase is observed in the occipital, parietal, and temporal lobes during a short-term memory task [72, 73]. The ongoing EEG oscillatory rhythm in the higher frequency is considered in [69] as a correlate of high-speed WM comparison during the recall (see Figure 4).

Following the above neuroscience findings, our main hypothesis is that the ongoing EEG oscillatory rhythms during top-down visuospatial selective attention show specific evoked bursts in higher frequency bands and electrode positions and are functionally connected in different ways during attentional states compared with passive view.

### 4.2. Participants

Data were collected from 11 healthy participants (3 females) with normal vision, all right handed, and with mean age 33.09. The EEG session lasted for half an hour in total. All participants signed informed consent before the experiments.

### 4.3. Stimulus Presentation

Participants were seated in front of Dell laptop with a 14-inch flat screen monitor with a resolution of 1366 × 768 pixels. So called Porteus mazes (https://www.mazes.ws/mazes-hard-puzzle-one.htm) were displayed on it, although any other website for playing hard mazes online can be used. An identical cable mouse had been used from all participants. A Python script is used for detecting whether the mouse is moving or not.

### 4.4. Method of Registration

In Section 3, the proposed fuzzy shell for developing a custom EEG BCI was implemented for studding the neuronal activity during the top-down visuospatial selective attention and the information processing during navigation. We tested different hypotheses during solving a Virtual Maze Navigation Task (VMNT) and proved the concept in [74] that VMNT is well suited to evoke brain *γ* responses. We examined the brain activity during spatial exploration, path planning, and navigation, which rely on forming cognitive maps (CMs). According to Tolman [75], CMs enable one to get, encode, store, recall, and decode information about the relative locations in their everyday or symbolic spatial environment. Therefore, many top-down attentional systems participate during the spatial navigation in order to gather information and evaluate the options: attention to sensory observations over multiple spatial locations, attention to mental representation of paths with their temporal order, and attention to encode and retrieve information from WM or visual short-term memory.

### 4.5. Experimental Task and Trial Setup

In the present experiment, participants had to solve virtual hard mazes # 14th and #15th. A maze #13th was used as an exercise to make them use the laptop, mouse, and software.

The experimental VMNT task responses in three conditions are as follows:

- Condition 1: forming of cognitive maps, top-down visuospatial selective attention underlying spatial exploration, path planning, mental navigation, and evaluation of options, as well as encoding and storing the cognitive map in WM
- Condition 2: memory-guided visuospatial traversing, high-speed WM comparison during recall and decode routes for traversing
- Condition 3: instant visuospatial traversing, instant spatial exploration when the path is not bottleneck in the neighborhood or trial and error guided the route traversing

The mouse events (handled by a Python script in Node-RED) are used for identifying the current condition and depend on whether the mouse is moving or not. We assume that holding the mouse button presumes forming of CMs, while if the mouse coordinates are changing, the second or third condition occurred. We set two top-down visuospatial attentional conditions: forming of cognitive maps (FCMs) and visuospatial traversing (VST). We distinguish them according to the mouse events and saved the ongoing EEG oscillatory activity in terms of *R*_ERS^″^/ERD_*w*_^″^×*E*_ in different CSV files.

### 4.6. EEG Acquisition

EEG data was continuously recorded from neuroheadset “EPOC+” by EMOTIV Bioinformatics Company [76]. The recording sites from AF3, AF4, F3, F4, F7, F8, T7, T8, P7, P8, O1, and O2 were collected. The EEG signals then were preprocessed in frequencies from 4 to 50 Hz. EMOTIV EPOC + categorizes brainwaves by frequency into four main types: beta, alpha, theta, and delta using FFT. The FFT output is converted to power density (*μ* V^2^/Hz).

### 4.7. Experimental Protocol

At the beginning of the experiment, two baseline phases for the anterior and posterior cortex were performed. Electrode positions were topographically aggregated as frontotemporal cortex and parietooccipital cortex. In a passive view condition, the average power of frequency bands was recorded and after calculating the ERS” for the electrodes of interests the passive view baseline for each participant was set up.

Each trial starts with audio tones for 10 seconds and prompts the subject to listen to his/her brain and alerts that the upcoming maze solving task is starting. He/she is encouraged to click the left button of the mouse, which sets up the start of the trial and ERS” recordings for the two conditions, depending on the current mouse event. Time locked at the start of the trial auditory stimulation evokes bottom-up audio attention. This additional condition, bottom-up audio attention with passive view (AVA), is used as a neurophysiological indicator for bottom-up audio attention during amplitude-modulated tone in a range of 290 to 790 Hz. Mapping the corresponding frequency bands of interest to a specific Hz in the human hearing range of frequencies is evaluated whether the participant is stressed/excited or relaxed. The corresponding frequency in Hz, played by a PC player, correlates with the active brain rhythms: lower frequencies indicate ERS” in lower bands and higher frequencies indicate ERS” in high-speed bands, while the middle indicates modulation of bands power. Windows with length of 250 or 300 msec and a step of 125 or 150 msec with resolution 256 Hz are used. Depending on the length of the step, the latency is changed; e.g., the oscillatory rhythms in high *β* and *γ* bands are accessed each 125 msec, while the oscillatory rhythms in in *θ* and *α* bands are accessed each 150 msec.

The integration of the data streaming from the EPOC + headset to Node-RED is via a custom library of input nodes, EmotivBCI Node-RED toolbox [77]. The installation and the node descriptions are presented in [58]. Node-RED flows of how to design an example of the proposed BCIFS are uploaded in the Node-RED flow library to be shared with the community [78]. The first flow consecutively registers the EEG data from the headset for the electrodes and frequency bands of interest. The second flow initializes the linguistic variables, fuzzy sets, and performs the reference phase for the parietooccipital cortex in order to generate the membership functions. The third flow optimizes the parameters in the fuzzy membership functions by DT. The fourth flow initializes the fuzzy rules and performs fuzzy inference based on the chosen type of Sugeno-style aggregation. The last flow illustrates how to create a front-end graphical user interface.

### 4.8. Data Analysis

Event-related bursts in the average power of oscillatory rhythms relative to a preevent baseline period at the four frequencies and in EEG epochs are analyzed. The evoked ERS” were averaged across participants to produce a grand average in order to discriminate the bursts, important electrodes, and/or training the fuzzy membership function and FIS. First, we concatenate all experiments for the user electrode arrays (column vectors) into one big matrix and label them for the three conditions. Then, data is ready to use for post hoc interpretation of the results by statistical and ML models in MATLAB.

#### 4.8.1. EEG Data Analysis

The proposed BCIFS was used for the analysis of bursts in the average power of frequency bands of interest in each single epoch. EEG power has been labelled for each trial and in each epoch from the status of the mouse event. Thus, each training sample has a label associating the current condition of one single class *C*-visuospatial attention (*l*_*i*C_). The 1^st^ label associates the AVA condition; the 2^nd^ associates FCMs condition, while the 3^rd^ associates VST condition. The ERS” (bursts) over scalp site and bandwidth were evaluated in windows with a length of 250 (300) msec and a step of 125 (150) msec. These overall latencies are suggested for developers and are in line with [79] that reported short narrowband bursts (<150 msec) and the authors in [80] stated that a duration of “*γ* bursts” is 100–200 msec with similar duration of *θ* cycle. Only critical changes at source and scalp level are described in the fuzzy rules. The output is used to differentiate the condition. For instance, the next rule expresses the functional connectivity at temporoparietal level:

 
*IF* “*P*8_*T*_*ERS*”300”, “*high*”, “*T*8_*G*_*ERS*”250”, “*high*”, *THENl*_1C_,

where *P*8_*T*_*ERS* is a linguistics variable with fuzzy set “high.” Other options are “desync,” “low,” “ref,” and “artifact.” Artifacts that arise from either low device connectivity or blink/ocular/muscle movements showed ERS” with values over 150 units for low frequencies and 10 for higher. Artifacts were removed at reference phase and corrected during the test. *P*8_*T*_*ERS*”300 means that a positive-going *θ* power over a right-parietal electrode site displays maximum rate for the power increase with a peak latency of 300 msec.

**Figure 5:**
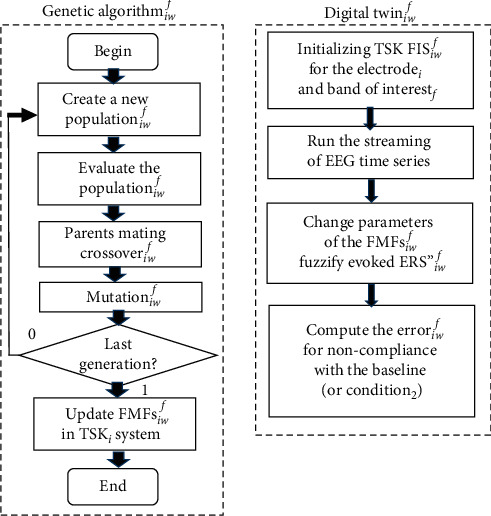
Flow diagram of the digital twin-based optimization procedure.

The membership functions for the fuzzy sets are built during the baseline phase:

(12) $$\begin{array}{c} \mu_{\text{Aref}}\left[\left(m-{\text{std}}^{*}c_{1}\right),\left(m-\text{std}\right),\left(m+\text{std}\right),\left(m+{\text{std}}^{*}c_{1}\right)\right], \end{array}$$

(13) $$\begin{array}{c} \mu_{\text{Alow}}\left[\left(m+\text{std}\right),\left(m+{\text{std}}^{*}c_{1}\right),\left(m+{\text{std}}^{*}c_{2}\right),\left(m+{\text{std}}^{*}c_{3}\right)\right], \end{array}$$

(14) $$\begin{array}{c} \mu_{\text{Alow}}\left[\left(m+{\text{std}}^{*}c_{2}\right),\left(m+{\text{std}}^{*}c_{3}\right),\left(m+{\text{std}}^{*}3c_{3}\right),\left(m+{\text{std}}^{*}3c_{3}\right)\right], \end{array}$$

where *c*_*i*_ are tuning parameters, *m* is the mean, and std is the standard deviation for 100 samples. The values of *c*_*i*_ can be predefined based on experience or obtained by an optimization procedure, such as genetic algorithm (GA) and particle swarm optimization. We first defined the parameters experimentally as *c*_1_ = 1.2, *c*_2_ = 1.4, and *c*_3_ = 2. Then, a digital twin-based optimization was designed and implemented based on the concept described in [81]. GA was used as a heuristics and is linked to the digital twin (DT). The population evaluation is performed in the DT with real EEG data in consecutive time windows. We constructed individual cost functions that define the individual error for each electrode and frequency band of interest.  Figure 5 illustrates the flow diagram of the digital twin-based optimization procedure, where GA _*iw*_^*f*^  and DT _*iw*_^*f*^  tune the fuzzy membership functions for the electrode *i*, window with a size *w*, and band *f* of interest, i.e., GA _*P*8 _^BH^  and DT _*P*8_^BH^ .

For each solution in the population _*iw*_^*f*^ , the fuzzy membership functions (12) and (13) in the fuzzy rules (15) and (16) are updated according to the new genes (tuning parameters) and each chromosome in the population is evaluated by the DT _*iw*_^*f*^ . GA _*iw*_^*f*^  minimizes the error by (17), which measures the difference between the simulated and real ERS”. The idea of the cost function is to compute the error for noncompliance with the already measured EEG oscillatory rhythms during the baseline phase. The fuzzy membership functions for the fuzzy sets “reference” and “low” (*μ*_Aref_ and *μ*_Alow_) should map adequately the dynamic of the BCI system in a passive view condition; i.e., *μ*_Aref_ should be 1 and *μ*_Alow_ should be 0. Thus, the temporal behaviour of the error within the several consecutive time windows serves as a cost function and after the last generation it should be minimum and close to zero.

(15) $$\begin{array}{c} R_{1}:\text{IF }x_{iw}^{f}\;is\;A_{\text{ref}}\text{ THEN }y_{1}=1-\mu_{A_{\text{ref}}}, \end{array}$$

(16) $$\begin{array}{c} R_{2}:\text{IF }x_{iw}^{f}\text{ is }A_{\text{low}}\text{ THEN }y_{2}=\mu_{A_{\text{low}}}, \end{array}$$

(17) $$\begin{array}{c} E\left(x_{iw}^{f}\right)=\frac{\mu_{A_{1}}\left(x_{iw}^{f}\right)*y_{1}+\mu_{A_{2}}\left(x_{iw}^{f}\right)*y_{2}}{\mu_{A_{1}}\left(x_{iw}^{f}\right)+\mu_{A_{2}}\left(x_{iw}^{f}\right)}, \end{array}$$

where, *i* ∈ {1,…, *e*′}, *e*′  is the number of electrodes of interest (*e*′ < *e*) and *f* is the frequency of interest for *i*-th electrode.

The cost function is described by TSK fuzzy rules of zero order (15) and (16), and the rules are aggregated and defuzzified by using the weighted average (10). Then, the GA receives the costs of the current population. *E* (*x*_*iw*_^*f*^) is 0 when the ERS” _*iw*_^*f*^  is in the baseline interval that results in a membership function close to 1 and by tuning the parameters *c*1, *c*2, and *c*3 in (12) and (13) the GA tries to minimize the cost function by selecting the best offspring after parents' mating and mutation. The optimization iterates to some ending condition; however, we found out that decent results could be obtained after only 10 generations with setting parameters: population size (the number of chromosomes in each generation) 40, parents mating 20, and 3 genes (the tuning parameters: *c*1, *c*2, and *c*3). The duration was about 2.5 minutes that did not cause users to become fatigued during the training stage. By analogy, the DT-based optimization was used to refine the coefficient *c*3 for the condition FCMs (forming of cognitive maps). During this training, the fuzzy rules *R*_3_ (18) and *R*_4_ (19) were used. In order to minimize the error by (17), *μ*_Ahigh_ (14) should be 1 and *μ*_Aref_ should be 0.

(18) $$\begin{array}{c} R_{3}:\text{IF }x_{iw}^{f}\text{ is }A_{\text{ref}}\text{ THEN }y_{1}=\mu_{A_{\text{ref}}}, \end{array}$$

(19) $$\begin{array}{c} R_{4}:\text{IF }x_{iw}^{f}\text{ is }A_{\text{high}}\text{ THEN }y_{2}=1-\mu_{A_{\text{high}}}. \end{array}$$

**Figure 6:**
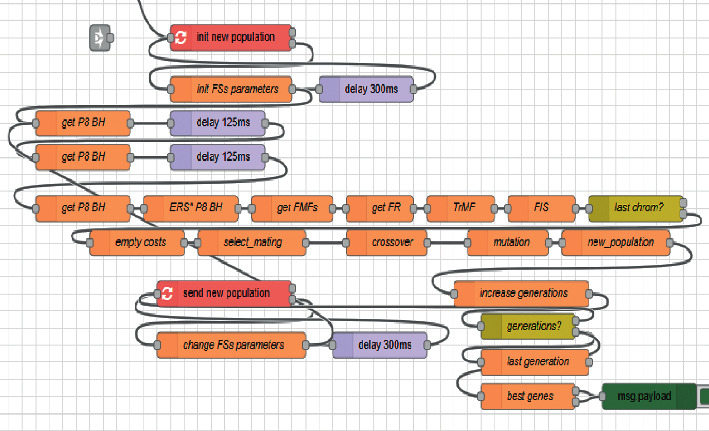
Digital twin-based optimization by GA implemented in Node-RED.

Since the coefficients slightly fluctuate for the different electrodes and bands, and across users, the coefficients typically were in the following ranges: *c*_1_ = [0.9 ÷ 1.3], *c*_2_ = [1.4 ÷ 1.6], and *c*_3_ = [1.9 ÷ 2.2]. The implementation in Node-RED is illustrated in Figure 6. The flow in JSON format is available in the Node-RED library [78].

After tuning the parameters of the fuzzy sets, the FRB was set up to test the three conditions. The following FRB is an example of how to test the EEG oscillatory rhythms correlating with label 1 with contradictions in assumptions:

 
*FR*1: *IF* “*T*7_*A*_ *ERS*”300” *is* “*high*” *THENl*_1C_
   *FR*14: *IF* “*T*7_*T*_ *ERS*”300” *is* “*high*” *and* “*P*7_*T*_ *ERS*”300” *is* “*high*” *THENl*_1C_
   *FR*15: *IF* “*T*7_*BH*_ *ERS*”250” *is* “*high*” *and* “*T*8_*BH*_ *ERS*”250” *is* “*high*” *THENl*_1C_

We analyzed more rules that mirror the different bursts in ERS/ERD during processing external audio inputs because the underlying band during audio stimulation is still strongly debated in the literature. Such rules that run simultaneously are *FR*11(*T*8*BH*); *FR*19(*P*6*G*); *FR*21(*F*7*G*); *FR*34(*F*8*T*); *FR*35(*T*7*T*); *FR*36(*T*8*T*); and *FR*39(*R*-*T*).

#### 4.8.2. Statistical Data Analysis

For statistical analysis of EEG data, the statistic package of MATLAB was used. An ANOVA compared ERS” for the band of interest on EEG scalp level between AVA, FCMs, and VST conditions. The statistical *p* value is commonly used to express the significance of research findings; however, according to the criticism in [82] that a single *p* value cannot meaningfully determine which pairs of means (groups) are significantly different for a given hypothesis, we use statistics and machine learning methods in MATLAB for multiple comparison based on Bonferroni approach to perform multiple *t* tests with statistically highly significant *p* < 0.01.

From the post hoc scientific hypothesis testing, the electrodes and frequency bands of interest are discriminated and irrelevant features discarded. Thus, the system designers reduce the high multidimensional input space (resulting from multichannel and frequency bands) in the antecedents of the fuzzy rules.

#### 4.8.3. Machine Learning Data Analysis

Since EEG power has been labelled for each trial and stimulus, we used supervised machine learning-based classification approaches. In order to classify the three conditions during the spatial navigation, we trained a fuzzy classifier proposed in [54] from the input-output data for ERS”. We slightly modify the criteria for the rule consolidation in order to keep all sparse data that are important when the bursts in the oscillatory rhythms are evaluated. During the consolidation stage, the rules are ranked not only by their strength (the number of samples they govern) but also by the global classification error of the rule. The number of iterations was 15, defined based on the consolidation stabilization (i.e., there are no more accepted transfers). The fuzzy classifier by RGRC was implemented by the single winner approach [51].

## 5. Results and Discussion

### 5.1. EEG Data

**Figure 7:**
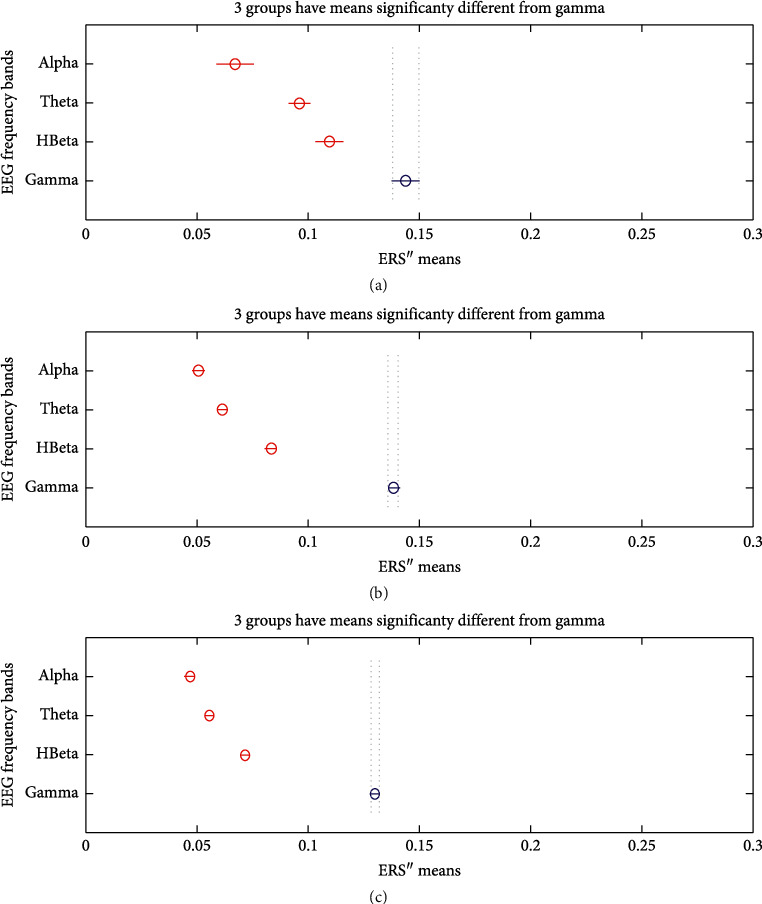
Multiple comparisons of ERS” means in frequency bands of interest. The blue points denote the statistically significance of *γ* band. (a) AVA. (b) FCMs. (c) VST.

**Figure 8:**
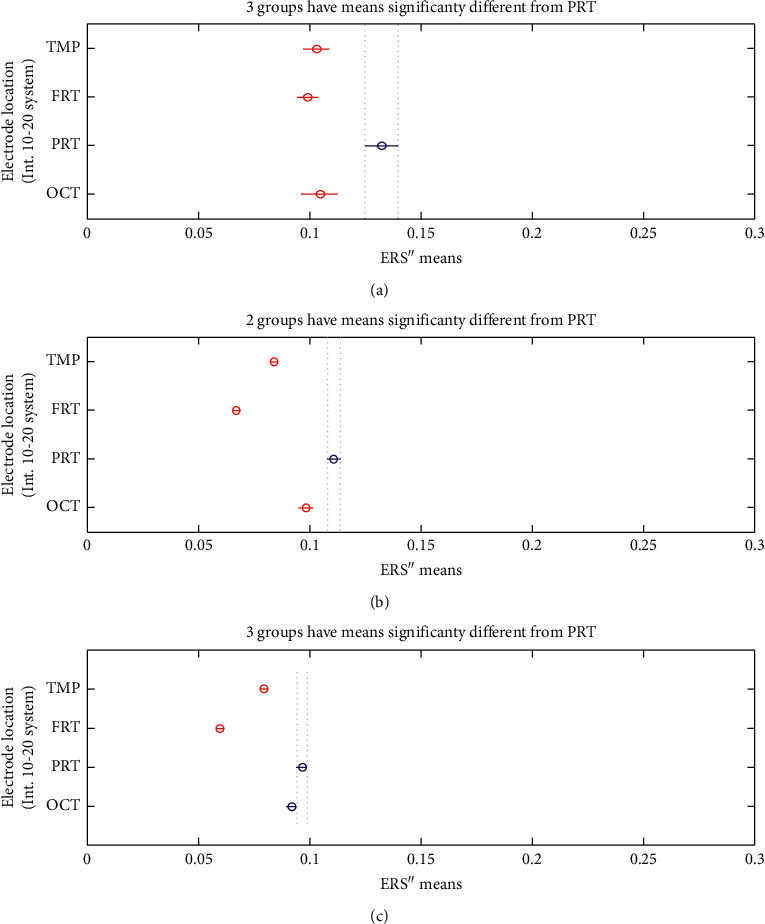
Multiple comparisons of ERS” means at scalp level. The blue points denote the statistically significance of the parietal site. (a) AVA. (b) FCMs. (c) VST.

**Figure 9:**
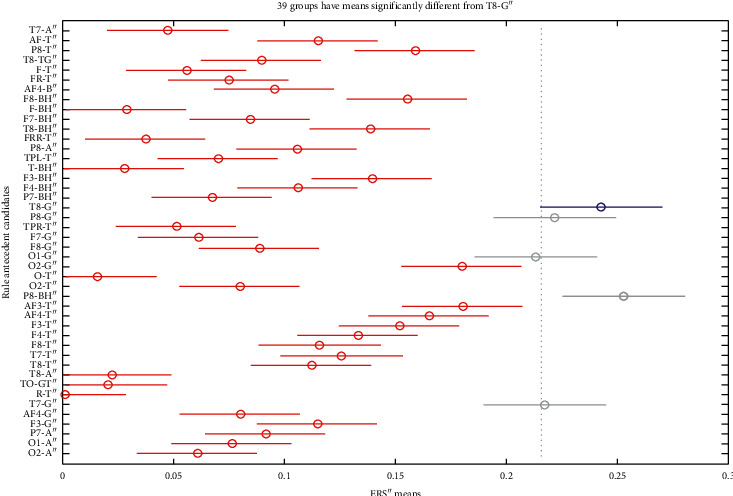
Multiple comparisons of ERS” means at source and site level for AVA. The blue points denote statistically equal ERS”. *γ* bursts of electrodes T8, P8, O1, and T7 are significantly different and important, as well as *β* burst of P8.

**Figure 10:**
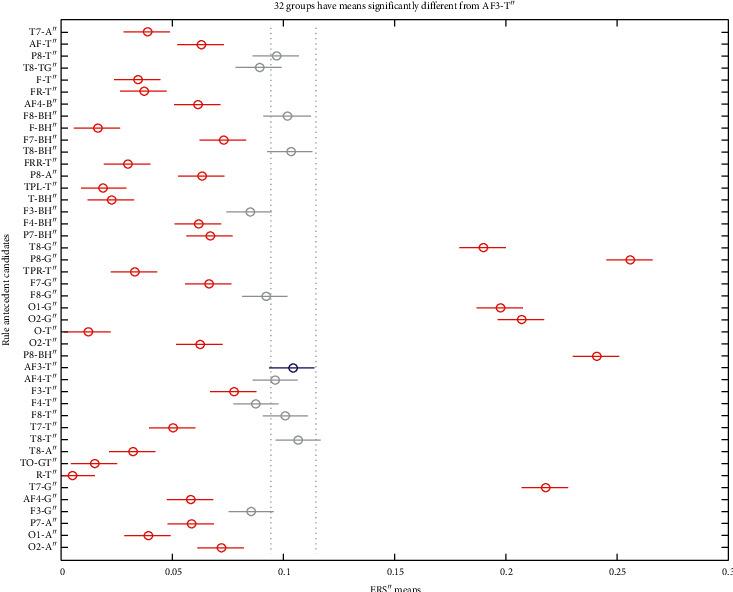
Multiple comparisons of ERS” means at source and site level for FCMs. The blue points denote statistically equal ERS” for *θ* bursts and electrode AF3 shows the highest *θ* burst.

The bursts in cut-off frequencies statistically were obtained by ANOVA and post hoc multiple comparison tests. ANOVA yielded a significant effect on the three conditions, showing an increase of ERS” in relation to baseline. VMST activated functional connectivity from frontal to parietal and occipital regions, as can be seen in Figures 7 and 8, reflecting the visual information processing and path finding processing with significant differences between the left and right hemispheres. The sites exhibiting high positive ERS” far exceeded the baseline at each frequency showing that the dominant is high-frequency *β* band (18–25 Hz) in the right parietal site (Figures 9 and 10). We explain this with the type of mazes that are “hard” and with discrimination difficulties. This is in line with the results in [83] claiming that the increased parietal *β* activity in the right temporoparietal region correlates with improving the perception of crowded stimuli. Similar results that a short narrowband burst of *β* waves correlated with memory and movement were reported in [79].

The detected high parietal *γ* activity is thought to reflect the visuospatial processing and ERS” increase with the difficulty of the task; thus, higher *γ* is associated with the FCMs condition (compare the values for *γ* bursts in Figures 7(b) and 7(c)). Other bursts in *γ* activity were observed in the temporal and occipital regions that were in line with studies reporting oscillatory activity during spatial navigation [68, 69, 84, 85]. Riddle et al. in [68] reported high *β* and *γ* in frontal and parietal cortex during visual search tasks. Herrmann et al. [84] proved that early evoked *γ* activity (50–150 msec) reflected allocating attention to a selected object and comparison with the templates in WM. Hong et al. in [69] proved that functional brain networks in *β* and *γ* bands were integrated in different ways during attentional state comparing to passive view state. Howard et al. in [85] showed that *γ* band power in the PFC increased directly and approximately linearly with WM load and this is in line with the decreased *γ* burst in Figure 7(c).

**Figure 11:**
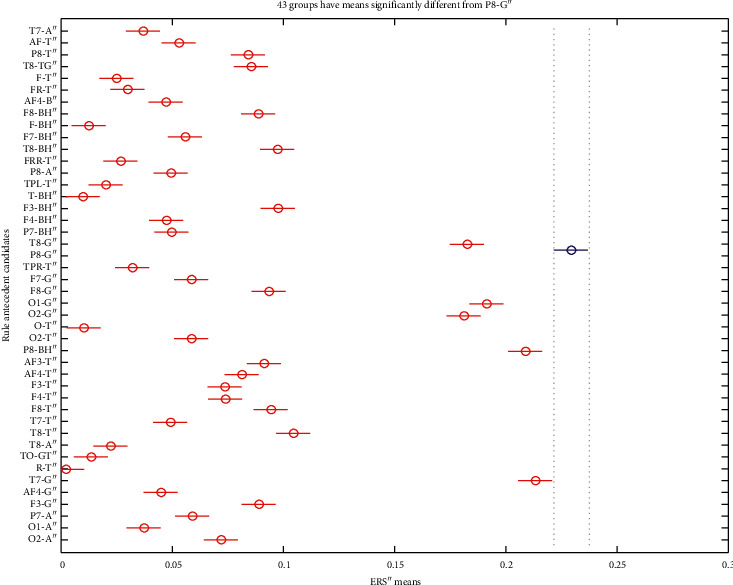
Multiple comparisons of ERS” means at source and site level for VST. The blue point denotes that only one *γ* burst of electrode P8 is significantly different and important.

In consistence with White [86], we localized functional connectivity of *θ* and *γ* bursts in right temporal and parietal regions during spatial navigation in both conditions. We gained this by designing fuzzy rules that consist of more than one linguistic variable to evaluate the coherence among the different electrodes and bands in one IF-THEN rule. This functional connectivity between *θ* and *γ* can be observed from *FR*4 (*T*8*TG*) in Figures 9–11. *FR*4 combines the linguistic variables for *T*8_*T*_*ERS*”250 and *T*8_*G*_*ERS*”250 with fuzzy sets “*high*.” Thus, this fuzzy rule has specific functional significance for the evoked neuronal assembly. ERS” shows high positive increase that far exceeded the AVA condition (*p* < 0.001). The ERS” for temporal *θ* and *γ* is higher in FCMs than in VST. This also can be seen in Tables 1 and 2 where *p* values of for *T*8*TG* are statistically significant (in bold). We explain this with increased task difficulties and corresponding working memory load that is in line with Meltzer et al. [87] who associated the increases in *θ* with *α* power as most prevalent in frontal midline cortex. Our post hoc multiple comparison with type of critical value “Bonferroni” (that rejects the null hypothesis at the 1% significance level) showed an increase ERS” in *γ* and increased frontal *θ* with increasing cognitive demand. *p* values (Table 2) that proved this were for the rules *AF*3*T*, *AF*4*T*, *F*4*T*, *FT* (frontal *θ*), *O*2*G*, and *P*8*G*. This is in line with Lisman et al. [88], who mirrored the effects of working memory load with *γ* power increases. He determined *θ*/*γ* coupling as a neural coding system beyond the hippocampus and most common in the occipital lobe.

We did not discover main frontal *θ* bursts that are not in contradiction with Caplan et al. [89], who reported that the dominant frequency they found during virtual maze learning occurred within *θ* band. We explained this with the findings in [72] that *θ* increased at the start of the encoding in WM and did not decrease until the end of a trial. In order to detect this activity, we need to evaluate also the first derivative, not only the second one. Meanwhile, we found *θ* bursts in the occipital and temporoparietal regions (see *p* values for *TPRT*, *T*7*T*, and *O*2*T*). The parietal *θ* showed significant differences in the left and right lobe between conditions. Also, ERS” for the parietal *θ* was higher in FCMs condition than in VST (Table 2: *p* values for *P*8*T*).  Figure 10 illustrates that almost all *θ* burst increased in ERS” and that the electrode AF3 shows the highest *θ* burst. This was in line with the results in [63, 72] associating *θ* rhythms with maintenance of stored information during the “retention” process.

*α* increased in the temporoparietal sites and had a clear lateralization with higher ERS” in parietal regions of the right hemisphere. This can be seen in Table 1 and Table 2 (*p* values for *P*8*A*) and is in line with results in [90] reporting the functional significance of EEG *α* power increases that are observed in various memory tasks and conflicting thinking.

After analyzing whether the audio stimuli evoked high ERS”, we confirmed the neuroscience findings in [91] that the low *γ* responses (40 Hz) were evident 80–120 msec after amplitude-modulated tone and were localized on the lateral right temporal region. We also observed high ERS” for *θ* and *γ*, as well as synchronization in the temporal regions. According to our results, the electrodes contributing to hearing are distributed along posterior right (T8, P8, and O2) and posterior left (T7 and P7) scalp sites.

### 5.2. System Performance

We designed a BCI system and translated the published neuroexpertise that correlates with sensory-evoked and event-related cognitive tasks in visuospatial navigation into 44 interpretable fuzzy rules. After averaging the data from the experimental sessions across all participants, the data was ready to use for post hoc interpretation of the results by different statistical or ML models developed in MATLAB.

The MATLAB scripts of how to average the data across all participants and how to perform the multiple comparison statistics can be seen in [92]. Based on the post hoc statistical analysis in MATLAB, we determined the candidate antecedents of significance.

We tested in MATLAB different fuzzy membership functions and the feasibility of several fuzzy, neurofuzzy, and fuzzy clustering approaches for post hoc evaluation of the neuronal activity and connectivity during top-down visuospatial selective attention. We developed in MATLAB the fuzzy rule-based classifier by RGRC (described in Subsection 4.8.3) and used membership functions in [54]. They are built upon two Gaussian curves defined by the positions of the peaks and standard deviations. The embedded Adaptive Neurofuzzy Inference System of Sugeno-type (ANFIS) in MATLAB [93] has been tested for training the membership function parameters. ANFIS combines the least-squares and backpropagation gradient descent methods. We considered the embedded fuzzy C-means (FCM) clustering in MATLAB [94], as well.

**Figure 12:**
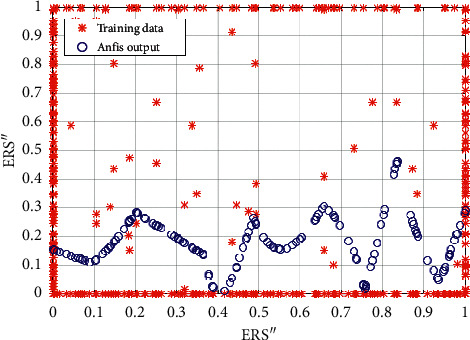
The trained fuzzy classifier in MATLAB with Adaptive Neurofuzzy IS (ANFIS).

**Figure 13:**
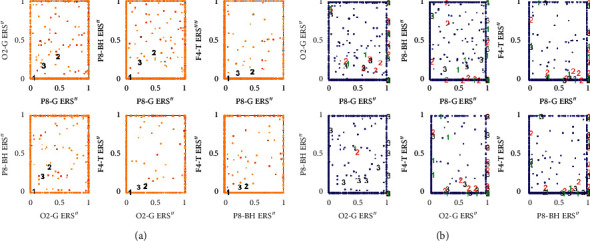
The trained fuzzy classifiers in MATLAB by (a) clustering or (b) metaheuristics. (a) FCM. (b) Fuzzy classifier by RGRC.

We trained these three fuzzy classifiers with the averaged numerical data from the experiments. The observations are as follows: (1) interval type-2 fuzzy membership function cannot be applied; (2) the ANFIS data did not match the training data even with an increase of the number of membership functions to 5 and the training epochs to 40 (Figure 12); (3) the RGRC showed better accuracy and was the most suitable for discriminating the burst in the oscillatory rhythms. The obtained results in Figure 13 can be compared with the FCM clustering in Figure 13(a). Probably, the classification accuracy of the used models depends on the properties of the dataset. ANFIS (Figure 12) and FCM clustering (Figure 13(a)) interpreted the sparse data like outliers, while the metaheuristic approach by RGRC (Figure 13(b)) evaluated and weighted the sparse data as specific information for functional connectivity.

In the future, the principles of transductive reasoning [95] will be tested in MATLAB by analogy to the proposed algorithm in [96]. It generates a local model at a single point of the workspace and for each new data to be processed the closest examples are selected from the known data. The main idea is to assign more importance (weight) to the specific information related to the data to be processed than to the general information provided by the entire training set [96].

By the experimental results, we confirmed the published neuroscience findings and provided causal evidence that the top-down visuospatial attention was mirrored in the oscillatory rhythms and *β* and *γ* rhythms had distinct functional roles. The found time locked presence of the ERS” at source and scalp level can be used as a general metric for interpretation of the spatiotemporal dynamics of the passive or evoked oscillatory rhythms. These results can be used for real-time attentional state classification in navigational tasks or for neurofeedback training of the top-down visuospatial attention.

## 6. Conclusion

A new brain state decoding is proposed that can be used as a feasible metric for interpretation of the spatiotemporal dynamics of the evoked neurooscillations. This new metric is exploited in the proposed BCI fuzzy shell for developing either passive or event-related BCIs, which can be used for control, monitoring, or research. The designed BCI works in IoT, in real time, and is device and service independent. The feasibility of the proposed BCI fuzzy shell was proven by real experiments. From them, we observed that *β* and *γ* bursts can be detected in real time and strongly believe in the reproducibility and ubiquity of the new proposed features that rate the increase of the evoked synchronization and desynchronization of brain rhythms at scalp level in response to time. The proposed BCIFS intends to support a wide range of EEG-based BCI applications and not a lot of skills in MATLAB programming and other software languages are required to support brain computations of the neuroscientists. Furthermore, the proposed software can be used for performing EEG experiments remotely, which is rather valuable nowadays.

## Figures and Tables

**Table 1:** Statistically high significant results (**p** **<** **0.01**) from the paired-sample *t* test of ERS” for each rule between condition 1 and condition 2.

T7A *p* = 0.16	AFT *p* < 0.01	P8T *p* < 0.01	T8TG *p* = 0.99	FT *p* < 0.01
**FRT** **p** **<** **0.01**	**AF4B** **p** **<** **0.01**	**F8B** **p** **<** **0.01**	**FB** **p** **<** **0.01**	F7B *p* = 0.16
**T8B** **p** **<** **0.01**	FRRT *p* = 0.15	**P8A** **p** **<** **0.01**	**TPLT** **p** **<** **0.01**	TB *p* = 0.27
**F3B** **p** **<** **0.01**	**F4B** **p** **<** **0.01**	P7B *p* = 0.95	**T8G** **p** **<** **0.01**	P8G *p* < 0.05
**TPRT** **p** **<** **0.01**	F7G *p* = 0.56	F8G *p* = 0.68	O1G *p* = 0.20	O2G *p* < 0.05
OT *p* = 0.24	O2T *p* < 0.05	P8B *p* = 0.37	**AF3T** **p** **<** **0.01**	**AF4T** **p** **<** **0.01**
**F3T** **p** **<** **0.01**	**F4T** **p** **<** **0.01**	F8T *p* = 0.11	**T7T** **p** **<** **0.01**	T8T *p* = 0.57
T8A *p* = 0.06	TOGT *p* = 0.20	RT *p* = 0.054	T7G *p* = 0.94	**AF4G** **p** **<** **0.01**
**F3G** **p** **<** **0.01**	**P7A** **p** **<** **0.01**	**O1A** **p** **<** **0.01**	O2A *p* = 0.16	

**Table 2:** Statistically high significant results (**p** **<** **0.01**) from the paired-sample *t* test of ERS” for each rule between condition 2 and condition 3.

T7A *p* = 0.55	AFT *p* < 0.01	P8T *p* < 0.01	T8TG *p* = 0.43	FT *p* < 0.01
**FRT** **p** **<** **0.01**	**AF4B** **p** **<** **0.01**	**F8B** **p** **<** **0.01**	**FB** **p** **<** **0.01**	**F7B** **p** **<** **0.01**
T8B *p* = 0.15	FRRT *p* = 0.30	**P8A** **p** **<** **0.01**	TPLT *p* = 0.50	**TB** **p** **<** **0.01**
**F3B** **p** **<** **0.01**	**F4B** **p** **<** **0.01**	**P7B** **p** **<** **0.01**	T8G *p* = 0.265	**P8G** **p** **<** **0.01**
TPRT *p* = 0.77	F7G *p* < 0.05	F8G *p* = 0.69	O1G *p* = 0.33	**O2G** **p** **<** **0.01**
OT *p* = 0.42	O2T *p* = 0.33	**P8B** **p** **<** **0.01**	**AF3T** **p** **<** **0.01**	**AF4T** **p** **<** **0.01**
F3T *p* = 0.34	**F4T** **p** **<** **0.01**	F8T *p* = 0.22	T7T *p* = 0.86	T8T *p* = 0.75
**T8A** **p** **<** **0.01**	TOGT *p* = 0.46	**RT** **p** **<** **0.01**	T7G *p* = 0.52	**AF4G** **p** **<** **0.01**
F3G *p* = 0.38	P7A *p* = 0.92	O1A *p* = 0.61	O2A *p* = 0.95	

## Data Availability

The data used to support the findings of this study are available (in anonymized form) upon request submitted to Anna Lekova (a.lekova@ir.bas.bg).
