# Corrigendum to “A Fuzzy Shell for Developing an Interpretable BCI Based on the Spatiotemporal Dynamics of the Evoked Oscillations”

**DOI:** 10.1155/cone/9842516

**Published:** 2025-08-11

A. Lekova and I. Chavdarov, “A Fuzzy Shell for Developing an Interpretable BCI Based on the Spatiotemporal Dynamics of the Evoked Oscillations,” *Computational Intelligence and Neuroscience* 2021 (2021): 6685672, https://doi.org/10.1155/2021/6685672.

In the article, there is an error in equation 2. The correct equation 2 is shown below:

Valence *=* *α F4/β F4* *−* *α F3/β F3*

We apologize for this error.

